# Septic Shock and Troponin I: Are They in a Relationship?

**DOI:** 10.5005/jp-journals-10071-23201

**Published:** 2019-07

**Authors:** Sachin Gupta, Deeksha Singh Tomar

**Affiliations:** 1,2 Department of Critical Care, Narayana Superspeciality Hospital, Gurugram, Haryana, India

## Abstract

**How to cite this article:** Gupta S, Tomar DS. Septic Shock and Troponin I: Are They in a Relationship? Indian J Crit Care Med 2019;23(7):294.

Sepsis is the leading cause of morbidity and mortality in patients admitted to intensive care unit (ICU). Cardiac dysfunction is present in nearly 70% of these patients and can present as hemodynamic instability, high cardiac biomarkers, arrhythmias or end organ damage.^1^ Cardiac troponins are recognized prognosticators in myocardial infarction. Troponins have been shown to be very sensitive and specific in predicting prognosis for patients with acute ischaemic stroke, pulmonary embolism and subarachnoid hemorrhage.^2,3^ This concept has now been extrapolated to prognosticate in patients with severe sepsis and septic shock, since there is no biomarker as yet which can accurately predict the outcome of patients with septic shock.

Many clinical and experimental studies in the past have shown that high troponins in septic shock patients indicate cardiovascular dysfunction and the associated poor outcome.^4^ The mechanism of rise of troponins in septic shock is still not clear but there are many hypotheses for the same. A demand-supply mismatch hypothesis, suggests presence of undiagnosed coronary artery disease patients with a rise in troponins during increased stress. Injury to cardiac myocytes by cytokines or endotoxins, focal myocardial ischemia, injury induced by free radical oxygen species produced by activated neutrophils and cardiac cell necrosis leading to regional wall motion abnormalities are other hypotheses.^5,6^

The clinical question is then, does elevated troponin I level indicate cardiac injury in septic patients? The studies in the past have shown that electrocardiography (ECG) and stress echocardiography and were normal in the patients with elevated troponin levels. The rationale was that microembolization of small vessels could lead to troponin release without ECG changes.^7^ Vallabhjosyula et al. conducted a study in which troponin T was measured at admission and at 3 hours. High admission troponin T was associated with greater severity of illness and serial measurements had no prognostic value.^8^ Many other small studies looked at correlation of troponin levels with outcome in septic shock with conflicting results.

In the issue of IJCCM, Jendoubi et al. studied the correlation of high sensitive cardiac troponin I (hs cTnI) with 28-day mortality in septic shock patients.^9^ They measured hs cTnI at admission and then at regular intervals upto 72 hours. The incidence of elevated hs cTnI was 47% in their subset. They concluded that the levels of hs cTnI was high at all intervals in non-survivor group and the presence of high hs cTnI at 72 hours has a strong correlation with 28 day mortality. The study brings out a few important facts. First, the incidence of increased troponin I is very high in patients with septic shock. Second, this is the first study showing a correlation of serial measurement of hs cTnI with 28 day mortality. The authors also highlighted the fact that the non-survivor group had elderly population as compared to the survivor group, indicating higher chances of myocardial injury as the age increases.

Cardiovascular dysfunction is a major reason for mortality in septic shock patients, and the presence of a novel biomarker which can accurately predict the 28-day mortality, can instead help in the treatment of such patients. This is promising but needs to be confirmed in multicentric trials. Till then, we can use high sensitive cardiac troponin I levels to prognosticate our patient outcomes.
